# Equitable Access to Genomic Molecular Testing for Australian Cancer Patients: Insights from the Victorian Precision Oncology Summit

**DOI:** 10.3390/curroncol31080337

**Published:** 2024-08-06

**Authors:** Genevieve Dall, Karen Harris, Nonie Chan, Stephen J. Luen, Sophia Frentzas, Daphne Day, Michelle Barrett, Anna Kilgour, Mark Buzza

**Affiliations:** 1 Victorian Comprehensive Cancer Centre Alliance (VCCC Alliance), Victorian Comprehensive Cancer Centre, Parkville, VIC 3052, Australia; genevieve.dall@vcccalliance.org.au (G.D.); michelle.barrett@vcccalliance.org.au (M.B.); 2 Monash Partners Comprehensive Cancer Consortium (MPCCC), Monash University, Clayton, VIC 3800, Australia; 3 Department of Medical Oncology, Peter MacCallum Cancer Centre, Parkville, VIC 3052, Australia; 4 Sir Peter MacCallum Department of Oncology, The University of Melbourne, Melbourne, VIC 3052, Australia; 5 Department of Medical Oncology, Monash Health and Monash University, Clayton, VIC 3168, Australia

**Keywords:** cancer, precision oncology, genomic testing, comprehensive genomic profiling, equitable access

## Abstract

The Victorian Precision Oncology Summit, convened in 2023, was a joint initiative between the Victorian Comprehensive Cancer Centre Alliance (VCCC Alliance) and the Monash Partners Comprehensive Cancer Consortium (MPCCC) and was proposed to guide a coordinated state-wide conversation about how the oncology sector can overcome some of the current obstacles in achieving equity of access to clinical cancer genomics for Victorian patients. Themes that emerged from discussion groups at the Summit include standardisation, centralisation, funding, education and communication and insights across those themes are outlined in this manuscript. The event served as a large consultation piece for the development of a broader precision oncology roadmap, which explores equitable access to molecular testing for Victorian patients, currently in development by the VCCC Alliance and MPCCC in collaboration with other key Victorian and national stakeholders. While this symposium was a Victorian initiative, it is felt that the insights garnered from this consultation piece will be of interest to consumer groups, clinicians, researchers, educators, policy makers and other key stakeholders in other states of Australia as well as in other countries implementing comprehensive genomic profiling within complex health systems.

## 1. Introduction

Genomic molecular testing for cancer driver aberrations has transformed cancer care, including diagnosis and treatment planning, and ranges from single gene tests to comprehensive whole genome sequencing (WGS). And yet, there are significant barriers to more widespread uptake of molecular testing. In Australia, some single gene and small gene panels are reimbursed by the federal government via the Medicare Benefits Schedule (MBS) [1,2]. However, cancer care is increasingly moving towards the use of comprehensive genomic profiling (CGP) in the form of large gene panels (such as the Illumina TSO500™ or Foundation Medicine assays) and WGS. Such tests are increasingly being used by Australian oncologists and haematologists to help guide treatment decisions but are currently not reimbursed and are only offered through research programs [1,2] or privately funded by individuals.

Uncertainty around which tests are reimbursed, where the tests are available, or even which tests are appropriate for a given patient, is preventing widespread adoption of CGP [3,4]. The large geographical expanse of Australia also represents an additional challenge to equitable access as CGP is mainly offered in metropolitan centres through research programs. Unless archival tissue is readily available, patients from rural and regional areas often must travel hundreds of kilometres to have the biopsies required for CGP.

Patient awareness and understanding of genomic molecular testing is another hurdle to greater uptake. A recent review of 21 studies assessing patient experiences and expectations of tumour multigene next-generation sequencing found that most patients have a poor understanding of molecular testing, with many confusing germline and somatic testing [5,6]. Fear of genetic discrimination by insurance providers or the perceived consequences of inherited genetic mutations to their family members have been identified as barriers to uptake of testing [6,7]. However, incidental germline mutations are rarely detected as part of CGP [8,9]. In Australia, genetic tests have no impact on eligibility for health insurance, and since 2019 there has been a partial moratorium in place restricting genetic test results from affecting life insurance products (although this is set to expire in 2024) [10]. Importantly, there have been limited efforts to address cultural or linguistic barriers for Aboriginal and Torres-Strait Islander and Culturally and Linguistically Diverse (CALD) communities accessing CGP [11,12].

The Victorian Precision Oncology Summit, convened in April 2023, was a joint initiative between the Victorian Comprehensive Cancer Centre Alliance (VCCC Alliance) and Monash Partners Comprehensive Cancer Consortium (MPCCC) and was proposed to guide a coordinated state-wide conversation about how the sector can overcome some of the current obstacles in achieving equity of access to clinical cancer genomics for Victorians. For the Summit, it was decided to focus on those tests that are not currently reimbursed by the MBS, such as CGP. The event also served as a large consultation piece for the development of a roadmap to explore equitable access to molecular testing for Victorian patients, currently in development by the VCCC Alliance and MPCCC in collaboration with other key Victorian and national stakeholders.

### Summit Organisation and Participants

The Precision Oncology Summit was co-convened by the VCCC Alliance and MPCCC, expanding the reach of the event across both networks. Collectively, these strategic alliances represent 20 research, academic and clinical institutions in Victoria with a common goal of improving outcomes for cancer patients. Representatives from both entities came together to hear expert presentations and to workshop ways to work towards a more equitable framework of access to molecular testing, with a specific focus on non-reimbursed molecular testing, or CGP. A Summit Steering Group was established at the start of the project, comprising 18 experts in the field, including 2 consumers.

The Summit was a hybrid event (online and in person) open to all and promoted across the VCCC Alliance and MPCCC networks. There were over 150 attendees (66 in person, 86 online) who were a mixture of medical oncologists, pathologists, nurses, researchers, molecular testing curators, industry, government representatives, education providers, consumers, and other health professionals. The geographical representation of attendees was 82% metropolitan, 6% regional, 11% interstate and 1% international.

## 2. Workshop Outcomes and Common Themes

After a series of keynote presentations from national and international speakers (including a consumer perspective), attendees of the summit separated into break-out groups to discuss six workshop topics (two in person groups and one online group per topic). Prior to the Summit, the Precision Oncology Summit Steering Group initiated a targeted scoping survey, disseminated via email to the Directors of Oncology and Haematology Clinical Services across Victoria, to ensure the program and discussion topics were needs-based (Appendix A). Respondents were specifically queried regarding their perspectives on enhancing accessibility to non-reimbursed molecular testing, thereby shaping the focal points of discussion at the Summit. The topics covered were: (1) access to non-reimbursed molecular testing, (2) molecular testing variability, (3) data collection, (4) molecular reporting and clinical utility, (5) clinician awareness and literacy, and (6) consumer awareness and literacy. Before the Summit, the attendees were provided with discussion guides summarising the topics and were asked for input on key questions that had been approved by the Summit Steering Committee.

Outcomes of the breakout discussions were synthesised and delineated into overarching themes that arose across the discussion topics, namely, 1. workforce education, 2. patient education and awareness, 3. standardisation, 4. centralisation, 5. funding, and 6. data and sharing (detailed below, and summarised in Table 1). While the insights garnered from the summit were extensive, it is acknowledged that there will be other key considerations (e.g., bioinformatics and the proper training of clinical bioinformaticians) that were not specifically discussed at the Summit but that would be important in the optimal implementation of CGP into complex health systems.

### 2.1. Workforce Education

Education of the cancer workforce is important for achieving equitable access to precision oncology. Providing clinicians with comprehensive education on molecular testing, including its applications, interpretation, effective patient communication, clinical decision making, and fundamental genetics and genomics, will enhance their confidence and competence in selecting appropriate tests. This education needs to cover the selection of molecular testing panels, understanding the diagnostic or therapeutic purpose of the test, and the limitations of different testing approaches. Significant strains in workforce education are associated with keeping abreast of this rapidly evolving field where genomics is being integrated into routine care and decreasing costs are increasing demands [13]. Challenges include a lack of a standardised curriculum, limited access to high quality resources and educators, limited interdisciplinary training opportunities, ethical and regulatory considerations, integrating complex content like data science, and maintaining sustainability.

#### Discussion Outcomes

The available resources for upskilling in precision oncology are characterised by a lack of formal guidelines, diverse providers with significant quality disparities, and a predominantly ad hoc and self-directed approach to learning. Upskilling in this area is often overwhelming for clinicians, and a systemic, more structured approach to guide health professionals on the best pathways for upskilling is needed. Such centralised education pathways could potentially be implemented through organisations such as the National Health and Medical Research Council (NHMRC) or Royal Australian College of Physicians (RACP), or by incorporating precision oncology as a formal degree requirement, as put forward by the Human Genetics Society of Australasia to integrate genomics and molecular testing into the training curriculum for clinicians, including pathologists and oncologists as one example; however, it was also highlighted that ongoing training through professional development would be required due to the highly dynamic nature of genomics. Ways to further increase engagement could involve utilising gamified online learning tools, applying genomic specific requirements within continuing professional development medical, nursing, and allied health frameworks, or providing focused sessions at conferences that start with the basics of precision oncology. Collaboration among hospitals across all regions is vital to share learnings and avoid fragmented pathways. Standardised guidelines, policies and practices should be implemented and publicised through dedicated healthcare communication channels.

The field of molecular testing is constantly evolving, and the discussion emphasised how challenging it is to determine what level of detail is relevant for various sub-specialities, along with the modality, format, and platforms that it will be delivered on. Who should author the educational materials was also discussed and it was noted that expertise and credibility should be prioritised, and the formation of a multidisciplinary subject matter team is crucial to provide authoritative guidance, content, and effective delivery methods. It is unreasonable to expect that all healthcare professionals will have a detailed, up to date knowledge of molecular testing; therefore, upskilling the majority in general knowledge (for example, knowing that tests exists and where to seek assistance), whilst providing specialised knowledge and skills for a limited number of professionals who require it may be more realistic and efficient. It is essential to carefully address sustainability measures to keep content current and readily accessible whilst avoiding creating isolated knowledge silos within the healthcare community.

Participants also recommended addressing cancer workforce skills shortages, particularly in data curation. With more molecular testing there will be a greater need for data curators with specialised oncology training. A significant lack of molecular pathologists specialising in the solid tumour space was also highlighted. Molecular pathology requires specific expertise, and the scarcity of professionals in this field, at least currently in Australia, hinders the widespread implementation of formal education programs and comprehensive workforce development.

### 2.2. Patient Education and Awareness

Consumer awareness of, and literacy in, molecular testing and its implications for their care is important for improving autonomy, advocacy, and patient outcomes. Improved understanding of which groups (e.g., different geographical groups, CALD groups, etc.) are not accessing precision oncology will be important so that communication can be tailored in an appropriate format to reach those most in need.

#### Discussion Outcomes

A strong link between consumer and oncologist was highlighted as being very important by discussion participants, a fact supported by the recent literature [14]. However, support networks and other allied health professionals were also suggested to ensure consumers feel empowered to ask questions and make informed decisions about how molecular testing could benefit their treatment. To achieve this, it was noted that clinicians (including primary care clinicians) and nurses need to better understand patient needs and have higher levels of awareness of the benefits of molecular testing and support to disseminate this information to patients. A recent review of patient experiences of molecular testing noted that patients prefer to discuss their molecular testing results with an oncologist or nurse, rather than receiving written, internet-based, or video-based resources to explain the findings [5]. Nurses as educators and pathway navigators was noted as being highly valued by Summit participants and could be a potential avenue for increasing patient education.

The role of the consumer as an advocate was also raised in the discussion groups. Consumer advocates provide an important avenue to disseminate information and inspire and educate others. Harnessing community connections could assist consumers in understanding the options available to them, along with providing support to access information from existing credible resources such as Cancer Australia and the Cancer Councils. Videos and blogs of consumer stories could be another way to help raise awareness. Providing consumers with a platform to advocate for issues important to them, and to help shape policy direction, is critical; however, consideration must be given to who should act in this role and how adequate representation across the spectrum of consumers could be achieved. Improved coordination of support groups, and the establishment of a consumer alliance, would be one way to facilitate this. Developing a clear, purposeful, and collaborative consumer advocacy strategy will be essential for maximising the impact of the consumer voice and for improving consumer awareness of the benefits of precision oncology. Considerations should include consultation with diverse populations, development of training and education programs for consumer advocates, and centralised coordination of advocacy groups to lobby for positive change more powerfully.

### 2.3. Standardisation

Developing standardised guidelines and frameworks is essential to provide clinicians with the confidence to order molecular testing for their patients. Consistency in terminology, reporting, and outcomes (as they relate to clinical significance and local clinical trial information) is crucial to mitigate against misinterpretation and improve the quality and reliability of the test results. Establishing quality benchmarking and optimal care pathways in precision oncology will be important for embedding genomics and precision oncology into routine clinical workflows and for improving equitable care for patients.

#### Discussion Outcomes

Variations in data collection practices were noted by Summit discussion participants as a major issue, as they give rise to disparate datasets that impede the effective exchange and analysis of information. This situation can be attributed primarily to the absence of standardised reporting mechanisms and a shared language specifically tailored for molecular testing [15,16]. Participants agreed that the current, National Association of Testing Authorities (NATA)-mandated minimum requirements are currently too broad, and inclusions such as mutations described using Human Genome Variation Society (HGVS) nomenclature, clinical significance tiered according to the Association for Molecular Pathology guidelines, and information about local clinical trials, were all recommended. Implementing standardised reporting and collection methods will be essential for ensuring consistency and interoperability across different molecular testing practices. Developing templates that capture relevant clinical and patient information, tailored to specific cancer streams or panel sizes, will enable more accurate and uniform data collection. A recent survey of over 100 Australian oncologists reported a recommendation to include therapy recommendations and gene rankings that are evidence-based in comprehensive genomic profiling reports as standard practice [17]. Establishing a shared language for data reporting will facilitate meaningful data exchange and analysis.

It was also suggested that promoting a closer network and collaboration among clinicians could enhance the understanding and utilisation of molecular testing. Establishing forums, conferences, and platforms for knowledge sharing and discussion would facilitate the exchange of best practices and improve the consistency and standardisation of testing approaches. Improving the awareness and accessibility of molecular tumour boards was also suggested as an enabler towards the greater equity of access to molecular tests.

### 2.4. Centralisation

Centralisation was a common theme that emerged across several topics at the Summit, with participants calling for centralised platforms of information relating to the molecular tests themselves, as well as around clinical trials, data interpretation tools and education resources.

#### Discussion Outcomes

The rapid rate of change within precision oncology is a barrier to clinicians maintaining up to date knowledge on the appropriate test, interpretation of that test and access to potential clinical trials. Access to a centralised resource of experts in precision oncology that can guide decision making and advise on access to treatments and trials, as well as a monitored, broadly available troubleshooting platform and/or decision tree are required to better support clinicians to provide equitable care for all cancer patients. The centralised approach to tissue typing [18] and the National Coronavirus Helpline [19] were identified as existing models that such a resource could be based on. This could also be achieved through a clinical fellow ‘on call’ through the VCCC Alliance and/or MPCCC that could provide personalised support, and regional “champions” skilled in precision oncology to support a given regional area. Molecular Tumour Boards were again identified as an important educational and peer-support tool. Currently, informal networks (such as instant messenger groups) are available to some clinicians, but it was noted that the ad hoc nature of this is perpetuating inequity.

In terms of the tests themselves, creating a centralised platform entity that integrates pathology services and outlines guidelines for specific tumour types may address the issue of fragmented laboratories. This platform could serve as a repository of information, including recommended tests, tissue type requirements, and turnaround times. Encouraging collaboration and cooperation among pathology laboratories through the establishment of pathology networks could help coordinate testing efforts and prevent fragmented and duplicative approaches. This integrated approach would facilitate the development of common structures, guidelines, and quality assurance measures for molecular testing.

Establishing a central registry through data linkage would enable more comprehensive data aggregation and analysis, support population-level research, and provide a broader perspective on molecular testing outcomes that could drive future innovation. A centralised registry would reduce the risk of health data loss when patient care transitions between different healthcare providers.

A barrier to centralisation identified across different discussion groups was that databases are not well maintained and can be difficult to navigate. Development of a centralised website, other online resource or an application that brings together all local information about available genomic tests and clinical trials and mandates the inclusion of clinical trial information would likely be a strong value-add. More regular updates for new available clinical trials and harnessing artificial intelligence to better maintain websites were noted as potential ways to support currency of information.

### 2.5. Funding

Developing a cost-effective centralised system and infrastructure is vital for equitable access. A collaborative effort between consumers, the health sector, and government should provide clear guidelines and procedures for accessing non-reimbursed molecular testing, ensuring transparency and equal opportunity for all individuals. Collaboration and financial support from government and industry could contribute to education, research, and resources, ultimately benefiting patients and advancing the field of molecular testing.

#### Discussion Outcomes

It was noted that health services need to work more closely with universities and research institutions to enhance access to non-reimbursed molecular testing. In the discussion groups (including both clinicians and consumers) there was a plea for there to be increased effort to change the perception of non-reimbursed molecular testing as being purely for research purposes, as outlined recently [2]. Partnerships (including joint funding) between government and industry may be the key to achieving more access to testing as well as off-label therapeutic options for some patients. Advocacy from both consumer groups and the health sector will play a vital role in making non-reimbursed testing a part of standard care and creating a centralised approach to testing, ensuring equal accessibility to resources for different organisations.

The discussion participants did note that a push to get CGP reimbursed will require overcoming many challenges. Implementing non-reimbursed molecular testing as the standard of care for all cancer patients raises questions about funding and scalability. It is important to determine who these tests will be funded by and how the results will be incorporated into patient care plans. This would need to link back to workforce education and how changes would be practically disseminated to clinicians. Inconsistent funding models limit the resources allocated to education initiatives. The absence of a single organisation responsible for the implementation of standardised education programs also adds complexity and requires coordination among multiple stakeholders. A collective impact approach [20] is required to reduce duplication and ensure the greatest efficiencies.

### 2.6. Data and Sharing

Data relating to non-reimbursed molecular testing, clinical utility, and patient outcomes in Victoria are not widely or uniformly collected or accessible for interrogation. Promoting data sharing among all stakeholders poses notable challenges. Presently, the absence of a clear linkage between data inputs and patient outcomes hinders the perceived benefits of data recording. Furthermore, mandating the collection of health data into a centralised repository will necessitate additional resources, thereby requiring a governing body to provide the necessary funding.

#### Discussion Outcomes

A significant barrier identified in the Summit discussion groups was the need to ensure data security and privacy when sharing molecular data across different healthcare institutions. Establishing secure data-sharing frameworks, data access controls, and compliance with relevant privacy regulations poses challenges that are important to overcome. Enacting legislation to standardise health data sharing was suggested by some participants as it would likely play a significant role in promoting data interoperability and privacy protections. Legal and policy frameworks would provide guidelines, requirements, and incentives for healthcare organisations to share molecular testing data while maintaining appropriate safeguards. Such legislation could facilitate more secure data exchange, enable research collaborations, and promote greater transparency.

It was felt that demonstrating how data sharing leads to improved patient care, informed decision-making, and scientific advancements would encourage stakeholders to actively participate in data collection and data sharing initiatives. Outcome data may also lead to increased investment from pharmaceutical companies [2] and other industry stakeholders who would be more likely to then support the sustainability and viability of data registries over the longer term. Governing bodies would also be able to track the progress of equity policies and frameworks. It was noted by participants that infrastructure such as the Victorian Cancer Registry already exists and could potentially be leveraged to include data pertaining to molecular testing that is already being collected, including through registry trials. Scoping work is required to identify existing resources that can be utilised, with the goal of avoiding duplication of data sets and the associated security risks that may arise.

Engaging patients as active participants in data collection processes, such as through patient-reported outcomes, was suggested by the consumer discussion groups. Such information would enrich the datasets, enhance patient-centred research, and promote patient empowerment. Self-reporting may also overcome disparities in health reporting, particularly between regional and metropolitan patients.

## 3. Next Steps

The VCCC Alliance and MPCCC are developing a collaborative Precision Oncology Roadmap that will provide a series of recommendations to address the current inequity of access to molecular testing based on the discussions held at the Victorian Precision Oncology Summit and subsequent consultation interviews with a range of key national stakeholders. These recommendations will have a focus on the Victorian health sector, in the context of, and complementing broader national programs including (but not limited to) initiatives such as the Cancer Australia National Cancer Genomics Framework, which is part of the Australian Cancer Plan, and the work led by OMICO [2].

Given the high level of engagement of a broad range of health professionals, data experts and industry partners at the Victorian Precision Oncology Summit, the early willingness of key stakeholders to be part of a more in-depth consultation process, and the well-established infrastructure and reach of the VCCC Alliance and MPCCC member and partner-base, Victoria is well placed to pilot initiatives that address key recommendations. Upon successful implementation, such pilots could be expanded nationally to improve equity of access to molecular testing for the benefit of all Australian cancer patients.

## Figures and Tables

**Table 1:** Summary of workshop and discussion group themes and outcomes.

Theme	Potential Actions
Workforce education	Establish systemic, structured approach to upskilling the workforce in precision oncology. Promote of collaboration and information sharing. Implement tiered learning (specialised versus general) in precision oncology.
Patient education and awareness	Target those most in need with communications. Empower consumers by building strong relationships and wide support networks. Develop a clear, purposeful and collaborative consumer advocacy strategy.
Standardisation	Establish quality benchmarking and optimal care pathways to support decision making. Standardise reporting and data collection to enable more effective data exchange and analysis. Establish widely accessible platforms for networking and collaboration.
Centralisation	Establish centralised, curated resources that provide up to date information. Integrate pathology services and information to minimise fragmented and duplicated approaches. Establish a central data registry to facilitate analysis of outcomes.
Funding	Develop a cost-effective centralised system and infrastructure for access to molecular testing. Link health services to universities and research institutes to enhance access to non-reimbursed testing. Establish a pathway towards non-reimbursed molecular testing becoming standard of care.
Data and sharing	Enact legislation to standardise health data sharing and provide legal and policy frameworks for secure data exchange. Develop impact data on the benefits of data sharing. Conduct scoping work to leverage existing resources. Engage patients as active participants in data collection process.

## Data Availability

Not applicable—no datasets are available.

## Appendix A

**Pre-Summit scoping survey (Audience: Oncologists/Haematologists)**
**Introductory text:**

Oncologists and Haematologists currently practicing in Victoria are invited to participate in a short, anonymous survey to help us understand how cancer patients access molecular testing within Victoria.

The survey consists of ≤14 simple questions, takes less than 5 min to complete and is entirely voluntary.

The results will be used to inform development of a roadmap towards equal access to molecular testing for all Victorian cancer patients and may be used for publication in the future.

Once the survey is complete, it will not be possible to withdraw your data as the survey is completed anonymously.

This survey is a joint initiative between Victorian Comprehensive Cancer Centre (VCCC Alliance) and Monash Partners Comprehensive Cancer Consortium (MPCCC). All data will be stored securely in accordance with organisational policies.

Please only proceed with the survey if you agree to these terms.

**The following questions relate to your service. If you attend multiple locations, please answer for your primary service.**

1. **Where is your service located?**
  a. Regional Victoria
  b. Metropolitan Melbourne
  c. Other (please specify)
2. **Is your service public or private?**
  a. Public
  b. Private
  c. Other (please specify)

**The following questions relate to *non-standard of care* molecular testing.**
**For the purposes of this survey, “molecular testing” is defined as either a genomic or gene expression assay (e.g., next generation sequencing panels, RNA sequencing), and “non-standard” is defined as any molecular testing assay that is not MBS-reimbursed. The following questions relate to *non-standard of care* molecular testing.**

3. **Do you order non-standard of care molecular testing for any of your patients?**
  a. Yes—*go to question 4 (blue questions)*
  b. No—*go to question 8 (orange question)*
4. **For which tumour streams do you refer patients for non-standard of care molecular testing (select all that apply)?**
  - Haematological cancers
  - Central nervous system cancers
  - Lung cancers
  - Melanoma and other skin cancers
  - Breast cancer
  - Colorectal cancer
  - Upper gastrointestinal tract cancers
  - Prostate cancer and other genitourinary cancers
  - Gynaecological cancers
  - Head and neck cancers
  - Sarcomas
  - Rare cancers
  - Cancers of unknown primary
  - Other cancers (please specify)
5. **How do your patients access non-standard of care molecular testing in your service (select all that apply)?**
  - Clinical trials (i.e., to assess eligibility for biomarker driven interventional trials)
  - Research programs (i.e., programs that provide molecular testing with or without a treatment intervention, e.g., MoST, ASPIRATION)
  - Local cancer centre NGS panels
  - Private pathology services based in Australia
  - Commercial assays based overseas (e.g., Foundation Medicine, MSK-IMPACT, Guardant Health)
  - Other (please specify)
6. **Approximately how many non-standard of care tests would you order in a given year?**
  - Less than 10
  - 10–30
  - 30–50
  - Greater than 50
7. **What is the typical source of funding for non-standard of care tests in your service?**
  - Patient-funded.
  - Externally funded (for example, through a clinical trial or research program at no cost to the patient)
  - Other (please specify):

***Continue to question 10***

8. **What do you think are the main barriers to widespread uptake of non-standard of care molecular tests? Select all that apply.**
  ▪ Lack of funding available
  ▪ Overall process too time consuming
  ▪ Turnaround time to receive results is too long
  ▪ Not readily accessible
  ▪ Lack of support interpreting data
  ▪ Lack of awareness of available NGS tests
  Lack of awareness of costs
  ▪ Lack of familiarity with NGS test scope
  ▪ No clinical utility
  ▪ The need to obtain patient consent for research-based testing
  ▪ Other (please specify)
9. **In your clinical practice, what is the primary reason you do not order non-standard of care molecular testing for your patients?**
  ▪ Lack of funding available
  ▪ Overall process too time consuming.
  ▪ Turnaround time to receive results is too long.
  ▪ Not readily accessible
  ▪ Lack of support interpreting data
  ▪ Lack of awareness of available NGS tests
  ▪ Lack of awareness of costs
  ▪ Lack of familiarity with NGS test scope
  ▪ No clinical utility
  ▪ The need to obtain patient consent for research-based testing
  ▪ Other (please specify)

***Continue to question 10***

10. **Do you attend, or have you ever attended, molecular tumour board meetings?**
  - Yes
  - No
11. **Please comment on any measures you think could be important towards improving access to non-standard of care molecular testing for patients across Victoria. Some examples could include education, infrastructure, standardised pathways of access and turnaround times.**
12. **Do you have any further comments about non-standard of care molecular testing you would like to share (optional)?**

**The following questions relate to *standard of care* molecular testing.**
**For the purposes of this survey, “molecular testing” is defined as either a genomic or gene expression assay (e.g., next generation sequencing panels, RNA sequencing), and “standard” is defined as any molecular testing assay that is MBS-reimbursed.**

13. **Do you (or the treating team) order standard of care molecular testing for any of your patients?**
  - Yes—go to question 14 (purple questions)
  - No—Why not? [To pop up].
14. **For which tumour streams do you refer patients for standard of care molecular testing?**
  - Lung
  - Melanoma and other skin cancers
  - Breast
  - Colorectal
  - Prostate cancer and other genitourinary cancers
  - Gynaecological cancers
  - Other (please specify)
15. **Approximately how many standard of care tests would you order in a given year?**
  - Less than 10
  - 10–30
  - 30–50
  - Greater than 50
16. **Do you have any further comments about standard of care molecular testing that you would like to share (optional)?**
